# A Fragment

**Published:** 1893-01

**Authors:** 


### A FRAGMENT.

When man in his primeval days,
The glory of the night beheld
The stars innumerable that filled
With golden light the whole of space;
The moon that shone a garish queen,
Amid the splendor of the scene,
Which rivalled e'en the sunlit glow,
That wakes to smiles the world below,
What wonder is't that he should hold
All these subservient to his world,
As he opined, a boundless plane
Of land and stream and surging main:
And reasoning thus of Nature's laws,
And holding fast his crude belief,
What wonder he, the primal cause
Ascribed to some almighty chief,—
Sovereign of earth and air and sky,
Invisible to mortal eye?
The rude conception of his mind,
To superstition yet inclined?
And being in his proud conceit
Of entities the most complete,
In his own image made he Him,
With wondrous reach of sense and limb,
And every passion to him known,
Intensified and overgrown:
A monster man in all his ways,
With lust of power and greed of praise,
Whose will was law, and whose decree
Of good or ill to humankind,
An everlasting destiny.
But wonder is't that man should hold,
Of Nature's God from age to age,
The self same concept as of old,
Nor priest, philosopher nor sage,
The meaner attributes recall,
Of the Great Father of us all.

—La Croix.

